# AT THE INTERFACE OF THE BRAIN AND ENVIRONMENT: DETERMINANTS OF COGNITIVE FUNCTION AND MOBILITY

**DOI:** 10.1093/geroni/igz038.1540

**Published:** 2019-11-08

**Authors:** Andrea Rosso, Michelle C Carlson

**Affiliations:** 1 University of Pittsburgh, Department of Epidemiology, Pittsburgh, Pennsylvania, United States; 2 Johns Hopkins Bloomberg School of Public Health, Baltimore, Maryland, United States

## Abstract

It is hypothesized that older adults are more influenced by environmental factors due to reduced tolerance to stressors and greater cumulative exposure. The role of environmental factors in brain and cognitive aging, specifically, has not been well studied. In this symposium, we highlight novel work at the interface of the environment and the brain in aging. The presentations assess varying aspects of the environment/brain interface within three cohorts. Two of the presentations focus on the environment as a risk factor for brain or cognitive aging. Sara Godina will present analyses of neighborhood socioeconomic status and neuroimaging markers of brain atrophy within the Health ABC Study, with the aim of understanding documented associations of neighborhood socioeconomic status with cognitive function. Jessica Finlay will present data from the REGARDS study linking weather conditions with cognitive changes. Climate influences social and physical activities and could thereby, impact cognitive aging. The brain also serves to process and integrate environmental data in order to direct behaviors in an adaptive manner. Two of the presentations assess how cognitive aging may impact individuals’ interactions with the environment. Using residential histories of participants in the Cardiovascular Health Study, Jonathan Platt will explore how cognitive trajectories change in relation to changes in residence. These analyses will demonstrate how residential changes may be either supportive or detrimental to cognitive aging. Finally, Andrea Rosso will present data from the Health ABC that demonstrates how cognitive aging may determine the vulnerability of older individuals to environmental influences on mobility declines.

